# Efficacy of *Duohuojisheng* decoction monotherapy for the treatment of knee osteoarthritis: A protocol of a systematic review of randomized controlled trials

**DOI:** 10.1097/MD.0000000000014510

**Published:** 2019-02-15

**Authors:** Zhi-ling Hou, Bo-yang Yuan, Ming-xia Fu, Lei Ni, Qiang Bao

**Affiliations:** a Department of Emergency Surgery, First Affiliated Hospital of Jiamusi University; b Department of Hematology, Jiamusi Hospital of Traditional Chinese Medicine; c Department of Chinese Medicine, First Affiliated Hospital of Jiamusi University, Jiamusi; d First Ward of Orthopedics Department, Inner Mongolia Xilin Gol League Hospital, Xilinhot, China.

**Keywords:** *Duohuojisheng* decoction, efficacy, knee osteoarthritis, randomized controlled trial, safety

## Abstract

**Background:**

This systematic review investigates the efficacy and safety of *Duohuojisheng* decoction (DHJSD) monotherapy for the treatment of patients with knee osteoarthritis (KOA).

**Methods:**

We searched relevant studies on DHJSD monotherapy for KOA from the databases of CENTRAL, EMBASE, MEDLINE, Cumulative Index to Nursing and Allied Health Literature, Allied and Complementary Medicine Database, Chinese Biomedical Literature Database, China National Knowledge Infrastructure, VIP Information, and Wanfang Data from the inception to January 1, 2019. Two researchers independently selected studies, collected data, and assessed the methodology quality by using Cochrane risk of bias tool.

**Results:**

This study evaluates the efficacy and safety of DHJSD monotherapy for KOA by assessing the pain intensity, stiffness, and disability of affected knee joints, and quality of life, as well as the adverse events.

**Conclusion:**

The results of this study provide latest updated evidence of DHJSD monotherapy alone for KOA.

**Ethics and dissemination:**

No ethical approval is required for this systematic review, because it is based on the published data, and not on individual patient data. Its findings is published in a peer-reviewed journal.

**PROSPERO registration number:**

PROSPERO CRD42019120405.

## 1 Introduction

Knee osteoarthritis (KOA) is a very common progressive joint disorder, especially in those aged 50 years and above.^[1–4]^ Patients with KOA often experience chronic pain, stiffness, swelling, movement restrictions of knee joint, poor quality of life, and psychiatric disorders.^[5–9]^ It has been reported that the prevalence of KOA ranges from 11.0% to 13.8% in Portuguese adults.^[10,11]^ In Chinese adult population, its overall prevalence rate is about 8.1%, with 17,128 Chinese residents aged 45 years and older.^[12]^ However, in the rural regions of China, the prevalence is about 16.57%, and the incidence is 29.25% for women and 24.71% for men aged 70 years and older.^[13]^

Treatments for this condition mainly focus on relieving knee pain, enhancing mobility and function, as well as improving the quality of life in patients with KOA.^[14,15]^ Traditional Chinese medicine (TCM), such as herbal medicine, acupuncture, moxibustion, Tai Chi, and so on, has been widely used to treat this condition in China.^[16–20]^ A variety of studies have reported to treat KOA by using Chinese herbal medicine, especially for the *Duohuojisheng* decoction (DHJSD).^[21,22]^ However, no systematic review has been conducted to assess the efficacy and safety of DHJSD monotherapy for the treatment of KOA. Therefore, this systematic review evaluates the efficacy and safety of DHJSD monotherapy for patients with KOA.

## 2 Methods

### 2.1 Eligibility criteria for study selection

#### 2.1.1 Study types

All the randomized controlled trials (RCTs) of DHJSD monotherapy for KOA are included without language and publication type restriction. However, non-RCTs, cluster RCTs, and nonclinical trials are excluded.

#### 2.1.2 Interventions

The therapeutic treatment applied in the experimental group is DHJSD monotherapy. Studies using DHJSD combination with any other interventions are not considered. The control group is any type of interventions, such as placebo, or medication therapy, except the DHJSD.

#### 2.1.3 Participants

Trials involving participants with KOA are included without restrictions of age, sex, race, and education background.

#### 2.1.4 Outcomes

The primary outcome is pain intensity, as measured by Visual Analog Scale, or Numeric Rating Scale, or any other relevant scales. The secondary outcomes are knee function and stiffness, as measured by Western Ontario and McMasters University Osteoarthritis Index or other scales, and quality of life, as measured by the 36-Item Short Form Survey or other instruments. Adverse events are also assessed.

### 2.2 Search methods for study inclusion

#### 2.2.1 Electronic databases searches

Two researchers independently and electronically retrieved following databases of Cochrane Central Register of Controlled Trials (CENTRAL), EMBASE, MEDLINE, Cumulative Index to Nursing and Allied Health Literature, Allied and Complementary Medicine Database, Chinese Biomedical Literature Database, China National Knowledge Infrastructure, VIP Information, and Wanfang Data from the inception to January 1, 2019. The detailed search strategy in CENTRAL database is shown in Table 1. Equivalent search strategy is built and applied to other electronic databases.

**Table 1:**
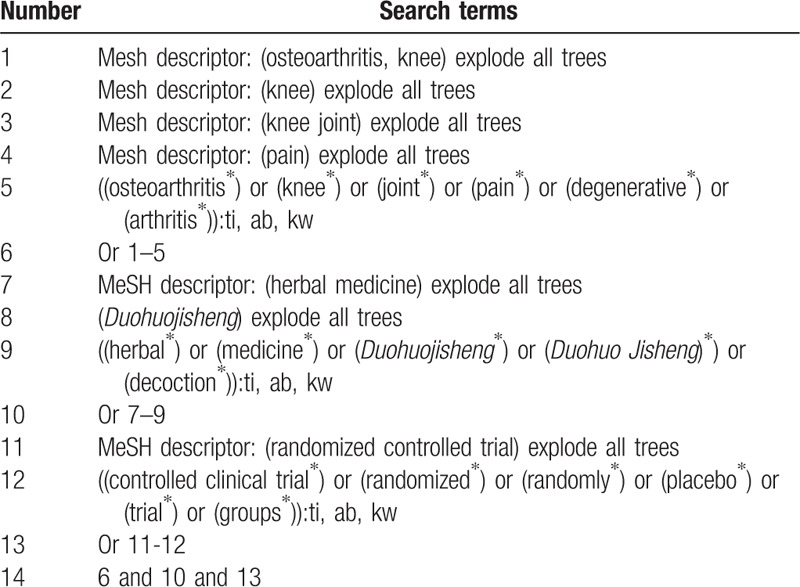
Search strategy applied in CENTRAL database.

#### 2.2.2 Searching other sources

Website of clinical trials registry and Google Scholar are searched. In addition, we manually searched the reference lists of included studies and relevant reviews to avoid missing any potential trials.

### 2.3 Data collection and analysis

#### 2.3.1 Study selection

**Figure 1:**
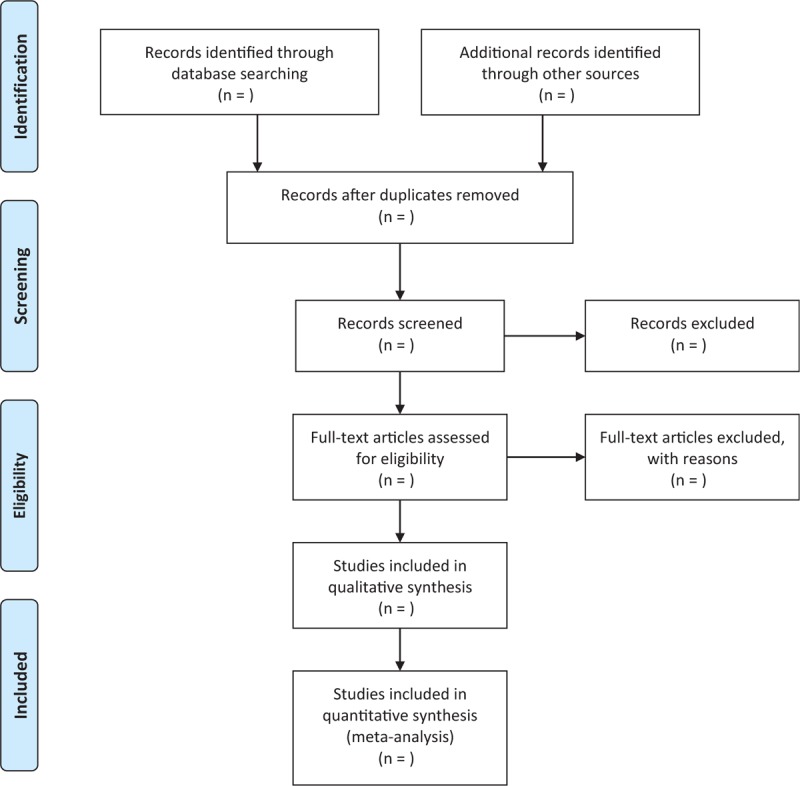
Flowchart of study selection.

All potential literature were uploaded to the Endnote X7 system. Two researchers independently conducted the selection and recorded their decisions based on a standard eligibility form, and PRISMA flowchart. Any disagreements about the inclusion and exclusion of the studies were settled through the discussion by a third researcher. Procedure details this study selection are presented in Figure 1.

#### 2.3.2 Data collection and missing data management

Two independent researchers read all included studies in full, collected the important information, and extracted the data via previous designed standardized data extraction form. The extracted information included general information (first author, published year, country, age, sex, funding, and setting), study methods (details of study design, sample size, randomization, concealment, blinding, insufficient reporting information, and any other potential risk of bias), interventions (period of treatment, follow-up, dosage, and types of treatments in both experimental and control groups), and outcomes (primary and secondary outcomes, and adverse events). The disagreements regarding the data extraction between the two researchers were judged by a third researcher through consultation.

If the data and essential information are insufficient or unclear, we will contact the authors from the primary trial to provide this information by email. If the missing data cannot be getable, then we will analyze the available data.

#### 2.3.3 Assessment of risk of bias

The two researchers independently carried out the assessment of risk of bias by using with the Cochrane Risk of Bias Tool. It consisted of seven domains, and each aspect was categorized into 3 levels: high, unclear, and low risk of bias. Any discrepancies were discussed by a third researcher involved.

#### 2.3.4 Measurement of treatment effect

For continuous outcome data, a mean difference or standardized mean difference with 95% confidence intervals is applied to present the extracted data. For dichotomous outcome data, risk ratio with 95% confidence intervals is utilized to measure the treatment effect.

#### 2.3.5 Unit of analysis

If the crossover studies are included in this systematic review, only the data of first phrase are used and analyzed. If included studies have multiple time point of outcome measurements, we combined the data into two term periods, including short term (within 5 weeks) and long term (over 5 weeks).

#### 2.3.6 Heterogeneity evaluation

Heterogeneity is detected by the chi-squared test. If *I*^2^ value is less than 50%, heterogeneity is considered as fair. Otherwise, it is considered as having significant heterogeneity among the included trials. Subgroup analysis is performed to explore the potentials factors of heterogeneity.

#### 2.3.7 Subgroup analysis

Subgroup analysis is carried out for investigating the possible causes of substantial heterogeneity in accordance with the different locations, patient characteristics, interventions, controls, and outcomes measures.

#### 2.3.8 Data synthesis

RevMan 5.3 software is employed for data synthesis and meta-analysis operation.

A fixed-effect model is applied to pool the data if *I*^2^ value is less than 50%. Otherwise, a random-effect model is used for data synthesis. In such situation, subgroup analysis is also conducted. If the heterogeneity is still significant after the subgroup analysis, then the data are not considered to be pooled, and a narrative summary is described instead.

#### 2.3.9 Sensitivity analysis

We carried out the sensitivity analysis to assess the robustness of studies based on the methodological quality, sample size, and missing data.

#### 2.3.10 Reporting bias

Funnel plot is carried out if there are more than 10 trials included. Meanwhile, asymmetry on funnel plot is also checked by Egg's regression and Begger's tests.

## 3 Discussion

KOA is one of most common conditions of orthopedic diseases. If this condition can be treated fairly and effectively, it may greatly affect the quality of life in patients with KOA. Numerous studies have reported that traditional Chinese can be utilized to treat this condition effectively, especially for the DHJSD monotherapy. However, no systematic review has addressed the efficacy and safety of DHJSD monotherapy for the treatment of KOA.

This systematic review firstly utilized rigorous methodology to assess studies reporting the outcomes of DHJSD monotherapy for KOA across all published RCTs. The findings of this study informed our understanding of the value of DHJSD monotherapy in treating KOA. This evidence may also be helpful to clinicians and patients.

## Acknowledgments

This work was supported by the Heilongjiang Provincial Health and Family Planning Commission Research Project (NO.2017-400). The supporter was not allowed to join in any parts of this study.

## Author contributions

**Conceptualization:** Zhi-ling Hou, Bo-yang Yuan, Qiang Bao.

**Data curation:** Zhi-ling Hou, Bo-yang Yuan, Ming-xia Fu, Lei Ni, Qiang Bao.

**Formal analysis:** Bo-yang Yuan, Lei Ni.

**Funding acquisition:** Zhi-ling Hou.

**Investigation:** Ming-xia Fu.

**Methodology:** Zhi-ling Hou, Bo-yang Yuan, Ming-xia Fu, Lei Ni, Qiang Bao.

**Project administration:** Zhi-ling Hou, Qiang Bao.

**Resources:** Bo-yang Yuan, Ming-xia Fu, Qiang Bao.

**Software:** Zhi-ling Hou, Bo-yang Yuan, Lei Ni, Qiang Bao.

**Supervision:** Zhi-ling Hou, Lei Ni.

**Validation:** Zhi-ling Hou, Ming-xia Fu, Lei Ni.

**Visualization:** Zhi-ling Hou, Ming-xia Fu, Lei Ni.

**Writing – original draft:** Zhi-ling Hou, Bo-yang Yuan, Qiang Bao.

**Writing – review & editing:** Zhi-ling Hou, Bo-yang Yuan, Ming-xia Fu, Lei Ni, Qiang Bao.
