# Efficacy of Duohuojisheng decoction monotherapy for the treatment of knee osteoarthritis: A protocol of a systematic review of randomized controlled trials: Erratum

**DOI:** 10.1097/MD.0000000000017484

**Published:** 2019-10-04

In the article, “Efficacy of Duohuojisheng decoction monotherapy for the treatment of knee osteoarthritis: A protocol of a systematic review of randomized controlled trials”,^[1]^ which appears in Volume 98, Issue 7 of *Medicine*, the third author name Ming-xia Fu should be Ming-xia Huo.
